# Identification of C2H2 zinc finger genes through genome-wide association study and functional analyses of *LkZFPs* in response to stresses in *Larix kaempferi*

**DOI:** 10.1186/s12870-023-04298-5

**Published:** 2023-06-02

**Authors:** Liying Shao, Lu Li, Xun Huang, Yanrui Fu, Da Yang, Chenghao Li, Jingli Yang

**Affiliations:** State Key Laboratory of Forest Genetics and Breeding, Northeast Forestry University, 26 Hexing Road, Harbin, 150040 China

**Keywords:** Genome-wide analysis, C2H2 zinc-finger genes, *Larix kaempferi*, Abiotic stress response, RT-qPCR

## Abstract

**Background:**

C2H2 zinc finger proteins (C2H2-ZFPs), one of the largest transcription factors, play a variety of roles in plant development and growth as well as stress response. While, the evolutionary history and expression profile of the C2H2-ZFP genes in *Larix kaempferi* (*LkZFPs*) have not been reported so far.

**Results:**

In this study, the whole genome of the *LkZFPs* was identified and characterized, including physicochemical properties, phylogenetic relationships, conservative motifs, the promoter *cis*-elements and Gene Ontology (GO) annotation. We identified 47 *LkZFPs* and divided them into four subfamilies based on phylogenetic analysis and conserved motifs. Subcellular localization prediction showed that most of the *LkZFPs* were located in the nucleus. Promoter *cis*-element analysis suggested that the *LkZFPs* may be involved in the regulation of stress responses. Moreover, Real-time quantitative PCR (RT-qPCR) results showed that Q-type *LkZFP* genes were involved in the response to abiotic stress, such as salt, drought and hormone stresses. Subcellular localization results showed that *LkZFP7* and *LkZFP37* were located in the nucleus, *LkZFP32* was located in both cytoplasm and nucleus.

**Conclusion:**

The identification and functional analysis of *LkZFPs* suggested that some *LkZFP* genes might play important roles in coping with both biological and abiotic stresses. These results could further increase understanding of the function of the *LkZFPs*, and provide some research direction and theoretical support.

**Supplementary Information:**

The online version contains supplementary material available at 10.1186/s12870-023-04298-5.

## Background

Zinc finger proteins (ZFPs), one of the largest transcription factor families in eukaryotes, are known for their ability to bind Zn^2+^ and their finger-like structure [1, 2]. The proteins contain a highly conserved “zinc finger” (ZF) domain, which is a stable three-dimensional structure consisting of different amounts of cysteine (C) and/or histidine (H) residues bound to zinc ions [3]. Based on the number and location of these residues, ZFPs are divided into ten types, including C2H2, C2C2, C3H, C3HC4, C2HC5, C4HC3, C2HC, C4, C6 and C8 [4]. ZFPs play a key transcriptional regulator in a number of biological processes in plants, such as hormone signal transduction, transcriptional regulation, trichomes and root hairs development [5].

C2H2 zinc finger proteins (C2H2-ZFPs), also called as TFIIIA-type or classical zinc finger proteins, account for a large proportion of zinc finger proteins currently studied [6]. The C2H2-ZFPs have two cysteines, two histidines and one Zn^2+^, which together form a tetrahedral structure containing an α-helix and two β-hairpins [7], among them Zn^2+^ guarantee the stability of the structure [8, 9]. The C2H2-ZFPs contain a characteristic motif composed of 25 to 30 amino acids, X2-C-X (2–4)-C-X12-H–X (3–5)-H, which has been widely found and verified in plants, animals and yeast [10, 11]. The C2H2-ZFPs include Q-type C2H2-ZFPs and C-type C2H2-ZFPs. The Q-type C2H2-ZFPs refer to C2H2 zinc finger protein containing highly conserved “QALGGH” sequence, which is unique in plants and does not exist in animals or yeast [12]. Any amino acid mutation of the “QALGGH” sequence could affect the DNA-binding ability of C2H2-ZFPs [13]. But the “QALGGH” sequence is not present in all C2H2-ZFPs, the C-type C2H2-ZFPs don’t have the conserved sequence. The C2H2-ZFPs can also be divided into four groups according to the form and number of zinc fingers, such as single-C2H2, triple-C2H2 (tC2H2), multiple-adjacent-c2H2 (maC2H2) and separated-paired-C2H2 (spC2H2) [14]. In addition, C2H2-ZFPs may have other functions with EAR motif. The EAR motif is hydrophobic and is thought to keep the zinc finger domain folded [15]. The most common types of the EAR motif are “LXLXL” and “DLNXXP” (where X represents any amino acid) [16, 17].

Since the first plant C2H2-ZFP was identified in *Petunia* and its expression was found to be tissue-specific and development-regulated [18], C2H2-ZFPs have been identified in numerous plants. As a transcription factor, the C2H2-ZFPs can effectively enhance plant tolerance to stresses such as low temperature, high salt and drought, by binding to specific promoter *cis*-elements of target genes [19]. For example, *AtSIZ1* enhances salt tolerance in *Arabidopsis thaliana* by reducing oxygen species (ROS) damage and osmotic stress and maintaining ion homeostasis through abscisic acid (ABA) signaling pathway [20]. *TaZFP1* and *MpZFP1* enhance plant tolerance to salt stress through a similar mechanism [21, 22]. Moreover, *MaC2H2s* may be involved in controlling cold stress in bananas by inhibiting the transcription of *MaICE1* [23]. In addition, transcriptomic analysis showed that nine typical *CsZFPs* in *Cucumis sativus* were significantly correlated with drought, low temperature, heat, and salt stress [24].

So far, the genome-wide analysis of C2H2-ZFP genes in higher plants has been reported widely: a total of 173, 109, 79 and 98 C2H2-ZFPs have been identified in *A. thaliana*, poplar (*Populus trichocarpa*), potato (*Solanum tuberosum*), grapevine (*Vitis vinifera*) [25–28]. However, C2H2-ZFPs has not been identified in *L. kaempferi*, even though *L. kaempferi* is an important ecological and economic afforestation species in Northeast China [29, 30]. The growth and development of larch are affected by various abiotic stresses, containing drought stress and cold stress. Therefore, the genome-wide identification of the C2H2-ZFPs gene family is very important to analyze and clarify their molecular function in *L. kaempferi*. In this study, we identified 47 *LkZFPs* and analyzed their physicochemical properties, phylogenetic relationships, conservative motifs, the promoter *cis*-elements, Gene Ontology annotation and subcellular localization. Since “QALGGH” sequence is critical to the DNA binding activity of C2H2-ZFPs, we first analyzed the expression pattern of Q-type *LkZFP* genes under salt, drought stress, ABA, methyl jasmonate (MeJA) and salicylic acid (SA) treatment by RT-qPCR. Our results enriched the structural information and expression pattern of *LkZFPs*, and provided a basis for investigating the role of C2H2-ZFPs in response to abiotic stress and hormone treatment *L. kaempferi*.

## Results

### Genome-wide identification of C2H2 zinc finger genes in *L. kaempferi*

After Blast alignment of C2H2-ZFPs in *Arabidopsis* and HMMER query, we screened these sequences manually based on “X2-C-X (2–4)-C-X12-H–X (3–5)-H” model and detected their structural domains. Finally, a total of 47 C2H2 zinc finger genes from *L. kaempferi* were identified in *L. kaempferi* genome and assigned from *LkZFP1* to *LkZFP47*. For the convenience of experimental analysis, the retrieved transcript ID was converted into gene ID. We recorded their detailed physicochemical information and subcellular localization results (Table 1). The number of amino acids ranged from 104 to 896, with an average of 384.61. The molecular weight ranged from 11.19 kDa to 98.49 kDa with the average 42.53 kDa. The isoelectric point (pI) ranged from 4.65 to 9.77. The value of GRAVY is negative and the instability coefficient is greater than 40, which means that most of *LkZFPs* are unstable hydrophilic proteins. The subcellular localization results of WoLF PSORT showed that *LkZFPs* was mainly located in the nucleus, and a small portion of *LkZFPs* might also be located in cytosol, chloroplast and mitochondrion.

**Table 1:** Physicochemical properties of *LkZFPs*

Gene name	Gene ID	Amino acid (aa)	Molecular weight	pI	GRAVY	Aliphatic index	Instability index	Subcellular localization
*LkZFP1*	Gene75	896	98,496.00	8.56	-0.706	62.97	56.71	nucleus
*LkZFP2*	Gene119	509	56,320.34	5.11	-0.735	61.14	78.75	nucleus
*LkZFP3*	Gene1729	354	39,312.04	6.47	-0.952	51.55	55.44	nucleus
*LkZFP4*	Gene3167	364	40,729.16	9.37	-0.995	45.36	59.28	nucleus
*LkZFP5*	Gene3302	236	26,155.67	5.53	-0.679	69.53	53.77	nucleus
*LkZFP6*	Gene3486	465	49,867.75	6.22	-0.805	45.87	62.98	nucleus
*LkZFP7*	Gene3666	465	49,965.90	6.44	-0.814	45.87	63.50	nucleus
*LkZFP8*	Gene3887	545	59,269.74	5.26	-0.611	62.35	53.56	nucleus, extracellular
*LkZFP9*	Gene4302	415	46,708.08	5.86	-0.780	72.22	53.32	nucleus
*LkZFP10*	Gene4583	532	59,835.33	4.72	-0.377	80.64	36.60	nucleus, chloroplast, cytosol, extracellular
*LkZFP11*	Gene4876	436	49,663.92	6.67	-0.960	57.68	57.44	nucleus
*LkZFP12*	Gene5984	415	46,708.08	5.86	-0.780	72.22	53.32	nucleus
*LkZFP13*	Gene6232	376	40,812.72	4.93	-1.038	51.09	36.61	nucleus
*LkZFP14*	Gene6261	332	35,632.61	4.72	-1.208	40.27	45.80	nucleus
*LkZFP15*	Gene6636	415	46,708.08	5.86	-0.780	72.22	53.32	nucleus
*LkZFP16*	Gene8600	373	40,970.29	6.49	-0.340	65.39	56.73	nucleus, peroxisome
*LkZFP17*	Gene14830	131	13,464.77	9.67	-1.381	26.11	35.10	nucleus
*LkZFP18*	Gene18263	526	58,062.71	5.64	-0.792	63.17	50.18	nucleus, chloroplast
*LkZFP19*	Gene19562	546	61,017.77	8.85	-0.528	80.51	54.98	nucleus, cytosol
*LkZFP20*	Gene19802	613	67,094.75	9.26	-0.760	55.45	57.11	nucleus
*LkZFP21*	Gene20506	545	60,904.62	8.85	-0.536	79.94	54.71	nucleus, cytosol
*LkZFP22*	Gene20604	542	59,738.74	6.14	-0.864	63.17	52.48	nucleus
*LkZFP23*	Gene20728	545	59,487.06	5.26	-0.588	63.76	52.13	nucleus, extracellular
*LkZFP24*	Gene21088	495	53,763.26	5.30	-0.688	61.35	56.20	nucleus
*LkZFP25*	Gene21903	576	63,418.10	6.57	-0.722	60.24	71.08	nucleus
*LkZFP26*	Gene22044	368	40,349.47	6.09	-0.636	66.28	56.44	nucleus
*LkZFP27*	Gene22325	495	53,739.28	5.30	-0.687	62.14	56.98	nucleus
*LkZFP28*	Gene23140	430	48,601.59	5.93	-0.633	70.58	57.22	nucleus
*LkZFP29*	Gene24195	358	39,567.44	5.84	-0.750	60.50	53.57	nucleus
*LkZFP30*	Gene24378	414	46,984.22	5.23	-1.168	47.39	48.86	nucleus
*LkZFP31*	Gene24812	377	41,158.34	4.96	-0.925	60.53	36.46	nucleus
*LkZFP32*	Gene25202	279	29,831.07	4.78	-1.348	35.34	41.14	nucleus, cytosol
*LkZFP33*	Gene26040	396	45,272.67	8.50	-0.636	74.60	36.86	nucleus, chloroplast, cytosol, extracellular
*LkZFP34*	Gene26098	413	46,904.14	5.49	-1.065	54.99	59.31	nucleus
*LkZFP35*	Gene26979	390	42,508.73	4.97	-0.995	53.26	36.89	nucleus
*LkZFP36*	Gene31551	328	35,706.63	8.52	-0.793	48.63	57.49	nucleus
*LkZFP37*	Gene32714	298	32,577.74	8.06	-0.530	59.16	47.21	nucleus
*LkZFP38*	Gene34994	248	27,996.82	4.65	-1.312	42.18	47.18	nucleus
*LkZFP39*	Gene35875	211	23,553.96	8.94	-0.591	80.57	63.92	nucleus
*LkZFP40*	Gene36016	259	29,835.67	8.85	-0.817	56.91	53.31	nucleus
*LkZFP41*	Gene36481	187	20,383.38	6.28	-0.689	57.43	41.46	nucleus, chloroplast
*LkZFP42*	Gene36582	265	30,993.00	9.03	-0.395	76.19	51.26	nucleus, extracellular
*LkZFP43*	Gene36664	197	20,651.80	4.67	-1.613	24.82	46.66	nucleus
*LkZFP44*	Gene37065	125	14,380.47	9.35	-1.029	56.24	62.53	nucleus
*LkZFP45*	Gene45065	165	19,472.75	6.63	-0.666	49.03	58.75	nucleus
*LkZFP46*	Gene45768	123	13,428.49	9.63	-0.925	57.24	26.01	nucleus, mitochondrion, cytosol
*LkZFP47*	Gene46308	104	11,192.96	9.77	-0.944	56.44	23.86	nucleus, mitochondrion, cytosol

### Phylogenetic analysis

**Fig. 1:**
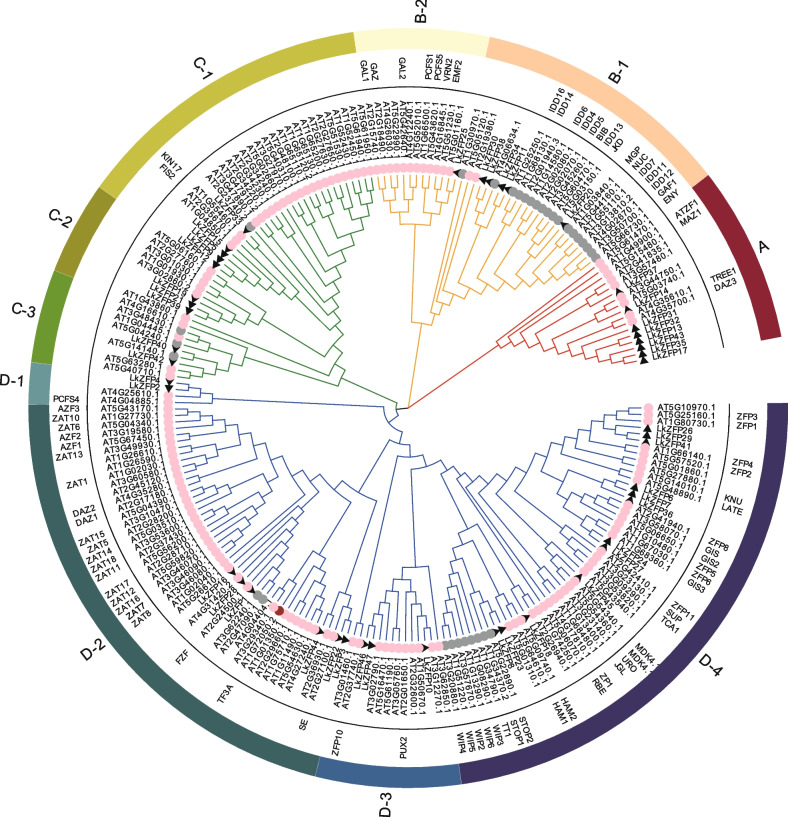
Phylogenetic tree of C2H2-ZF genes in *L. kaempferi* and *A. thaliana*. The phylogenetic tree was constructed by maximum likelihood method with 1000 times Bootstrap. The branches of the four subtribes are marked with different colors, and the 10 subgroups are marked with arcs of different colors outside the circle tree. The black triangle represents *LkZFPs*, and the circle represents *AtZFPs*. Gray, brown, pink represent *AtZFPs* in A, B, C respectively

The model plant *Arabidopsis thaliana* has been extensively studied, and the functions of many C2H2-ZFPs have been identified. Therefore, the phylogenetic tree of the C2H2-ZFPs of *L. kaempferi* and *A. thaliana* was constructed by the maximum likelihood method (Fig. 1), and the evolutionary relationship was further analyzed. According to sequence similarity and phylogenetic tree, these genes were divided into four subfamilies, with 17, 38, 47 and 118 members in subfamilies A, B, C and D, respectively. These four subfamilies could be further divided into ten subsets. The distribution of C2H2-ZFPs in *L. kaempferi* and *A. thaliana* was relatively uniform in the four groups, indicating that the genes of the two species were closely related. The adjacent parts of the phylogenetic tree may represent high homology.

### Q-type C2H2-ZFPs and EAR motif

**Fig. 2:**
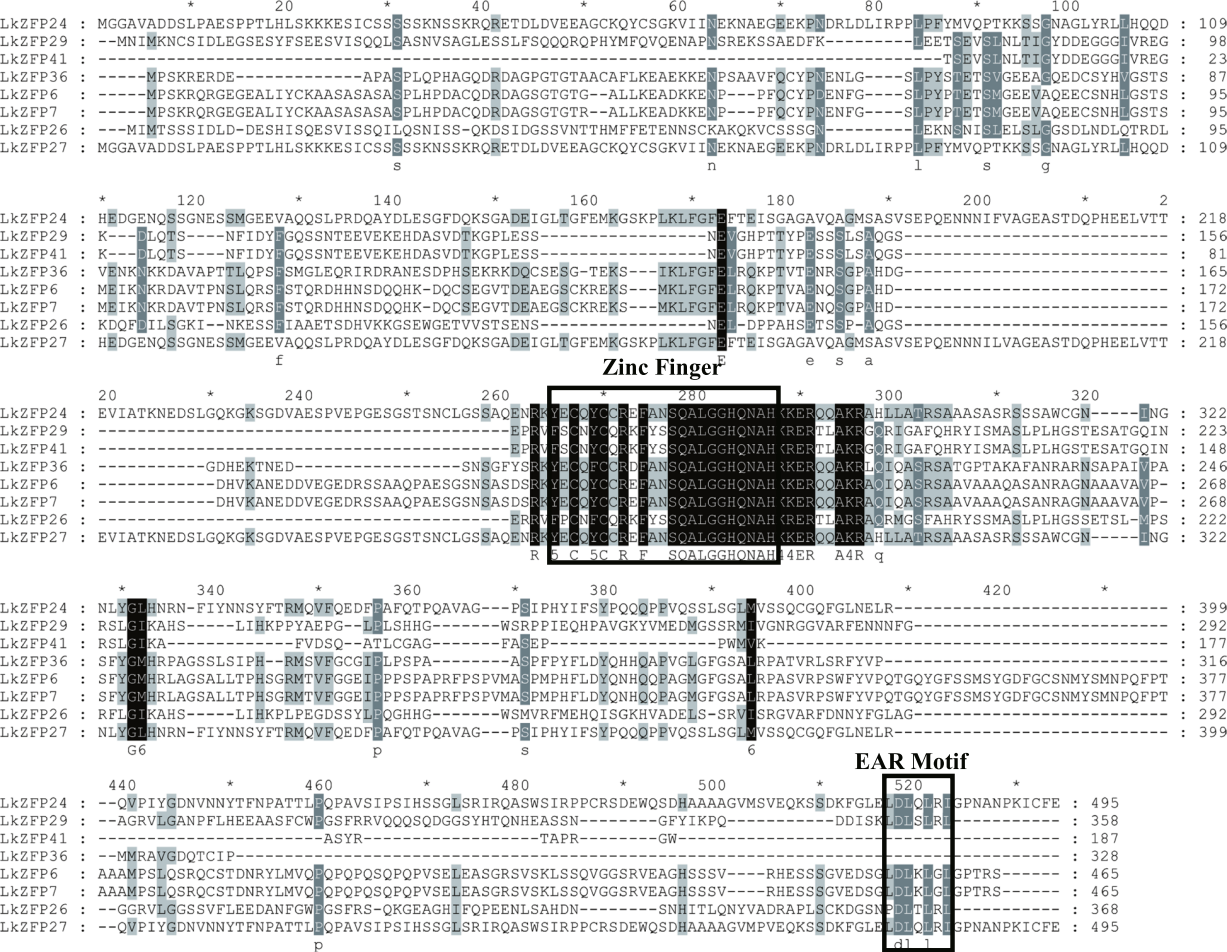
Sequence comparative analysis of eight Q-type zinc finger proteins in *L. kaempferi*. The black boxes represent the positions of conserved motifs

By analyzing the identified *LkZFPs*, we found that there were eight Q-type zinc finger proteins in subgroup D-4. We compared these amino acid sequences and marked the positions of the C2H2-ZFP conserved motif in the figure (Fig. 2). These *LkZFPs* contain the common zinc finger domain "X2CX2CX3FX3QALGGHX3H". During the comparison, it was found that six of the eight Q-type *LkZFPs* had the EAR motif “LXLXL” at C-terminus. The EAR motif has been identified as an activity suppressor gene [31, 32]. Therefore, we speculate that these six *LkZFP*s may have transcriptional inhibitory effects.

### Conserved motifs of *LkZFPs*

**Fig. 3:**
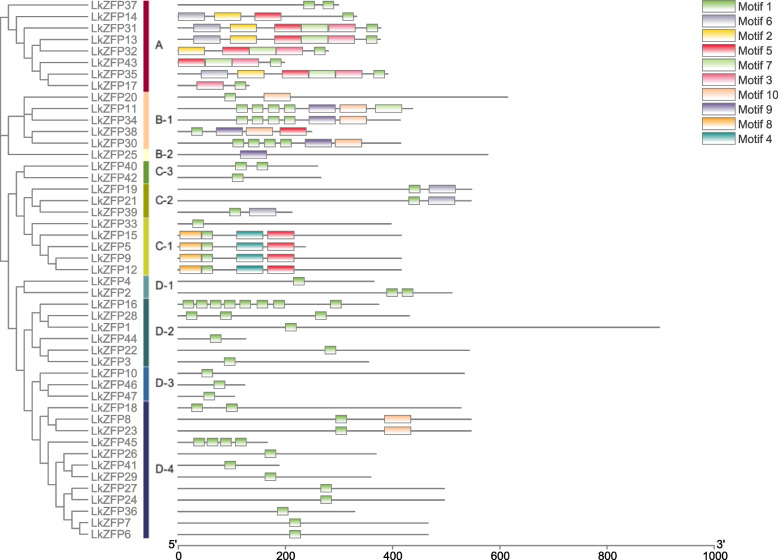
Phylogenetic tree and conserved motifs of *LkZFPs*. The phylogenetic analysis of *LkZFPs* protein sequences. Schematic diagram of conserved motifs of proteins were identified by MEME and corresponded to the name of phylogenetic tree. Each colored box represents a motif

**Fig. 4:**
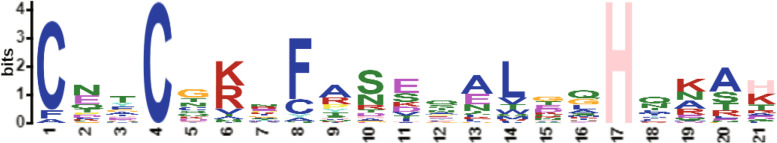
Sequence logos of the Motif 1 in proteins encoded by C2H2-ZFPs in *L. kaempferi*

We drew phylogenetic tree of *LkZFPs* separately (Fig. 3). In order to further analyze the diversity of conserved motifs, we used the tool MEME to retrieve 10 different motifs (Fig. 3). Motif 1 is distributed in almost all proteins. We suggested that Motif 1 (Fig. 4) may be considered as a conserved Motif of the *LkZFP*s. However, the protein motifs in subgroup D are less than others, which may be due to the poor similarity of genes and proteins. Motif 2 and Motif 3 only existed in subfamily A, Motif 9 only existed in subfamily B, Motif 4 and Motif 8 only existed in subgroup C-1, and other motifs were scattered in various subfamilies. As can be seen from the figure, proteins in the same subfamily have similar motif composition, indicating that their functions may have the similar functions, while proteins in different subfamilies may have different functions. Combined with the results of phylogenetic analysis, the reliability of classification is supported.

### Promoter *cis*-element analysis of *LkZFPs*

**Fig. 5:**
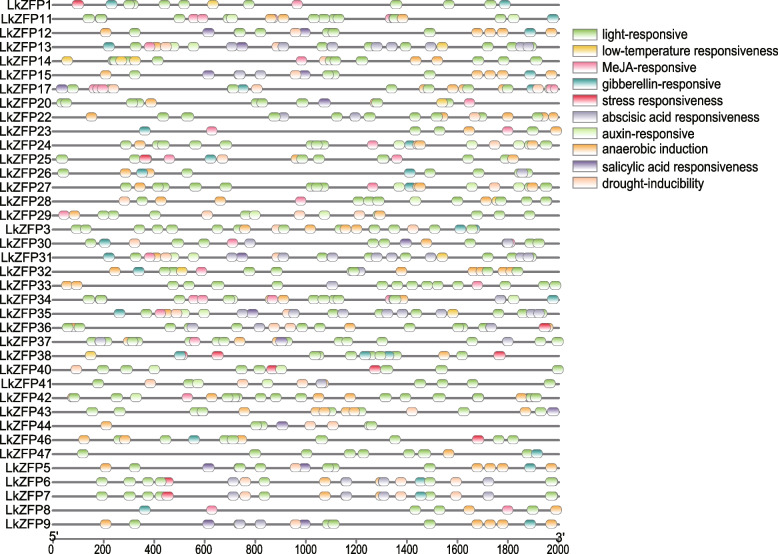
Promoters *cis*-element distribution of *LkZFPs*

When plants respond to abiotic stress, such as light, temperature and water, plants can regulate gene expression by inducing transcription factors to interact with corresponding *cis*-elements, so promoter *cis*-elements play a key role in the regulation of gene transcription. In order to better and intuitively understand the possible expression functions of the *LkZFPs*, we used PlantCARE to predict the cis-elements of the 2 KB promoter region upstream of the genes (Fig. 5).

We mainly extracted some stress response factors that have been widely studied. The predicted results showed that these promoter sequences contained multiple *cis*-elements, and most of them were involved in abiotic stress and plant hormones response, such as salicylic acid (SA), jasmonic acid (JA), auxin and abscisic acid (ABA). Among them, *cis*-elements related to light response are most widely distributed, including MRE (MYB binding site), Box II, AE-Box, G-box, GT1. There are many types of promoter *cis*-components, such as MBS (MYB binding site, involved in drought induction), ABRE (abscisic acid response element), ARE (anaerobic response element), TGA (auxin response element), TCA (salicylic acid response element), GARE and TATC-Box (gibberellin response element), LTR (low temperature response element), CGTCA and TGACG (jasmonic acid response element), and TC rich areas that can participate in the stress response. The presence of these promoter *cis*-elements is essential for plants to acquire the ability to adapt to abiotic stresses.

### Gene Ontology annotation of *LkZFPs*

**Fig. 6:**
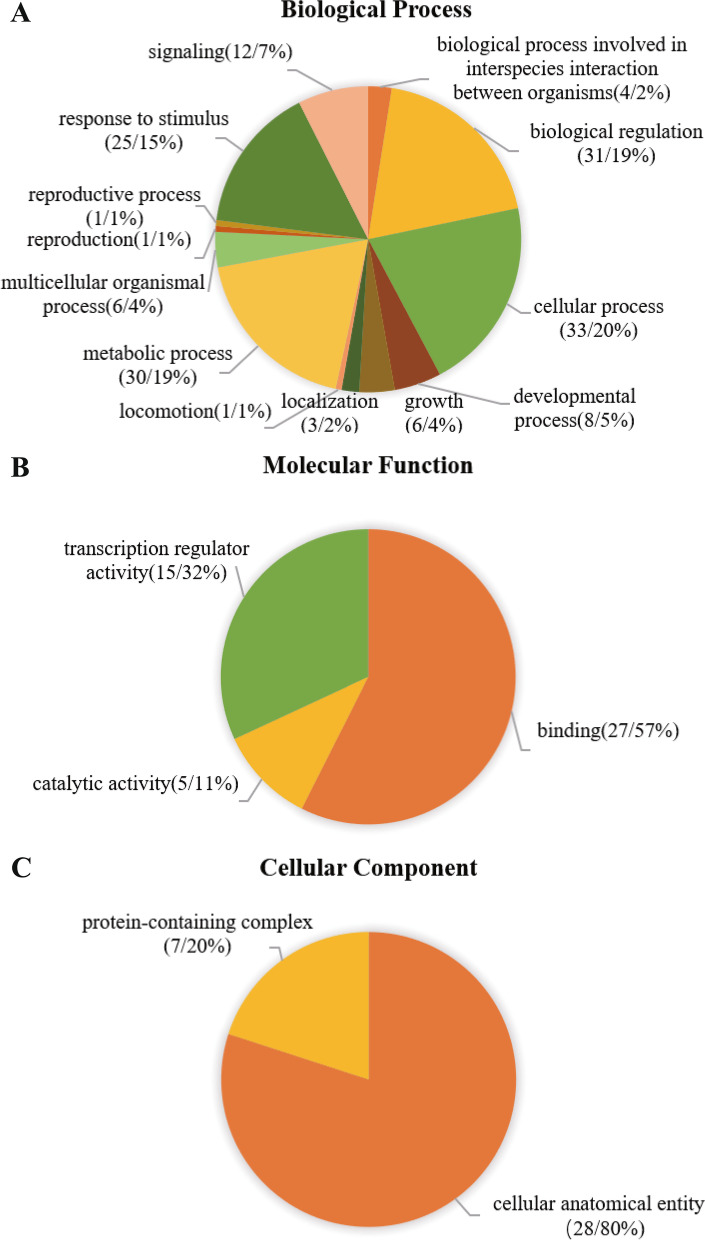
Gene Ontology (GO) results for *LkZFPs*

The biological processes, molecular functions and cellular components of the *LkZFPs* were studied analyzed based on Gene Ontology (GO) term assumption assignment (Fig. 6). The results indicate that *LkZFPs* may be involved in many biological processes. Of the biological process terms, some *LkZFPs* are predicted to play roles in the cellular processes (~ 20%), the metabolic processes (~ 19%) and the biological regulation (~ 19%), followed by the stimulus response (~ 15%). Molecular function prediction showed that more than half of *LkZFPs* were labeled as small molecules or/ion binding (~ 57%), which was consistent with the molecular role of C2H2-ZFP in DNA and metal ion binding. In addition, some *LkZFPs* were involved in transcription factor activity (~ 32%) and catalytic activity (~ 11%). The prediction of cell composition showed that most of *LkZFPs* were located in the cell (~ 80%) and others were located in the organelle (~ 20%).

### Expression pattern of Q-type *LkZFP* genes under abiotic stress and hormone treatments

Since “QALGGH” sequence plays an important role in the DNA binding activity of C2H2-ZFPs, we gave priority to the expression pattern analysis of Q-type *LkZFP* genes in this study. We used RT-qPCR to detect the relative expression levels of eight Q-type *LkZFP* genes in different tissues and different treatment times, so as to analyze their expression rules under different abiotic stress and hormone treatments. Studies have shown that these genes can effectively enhance plant tolerance to abiotic stress.

**Fig. 7:**
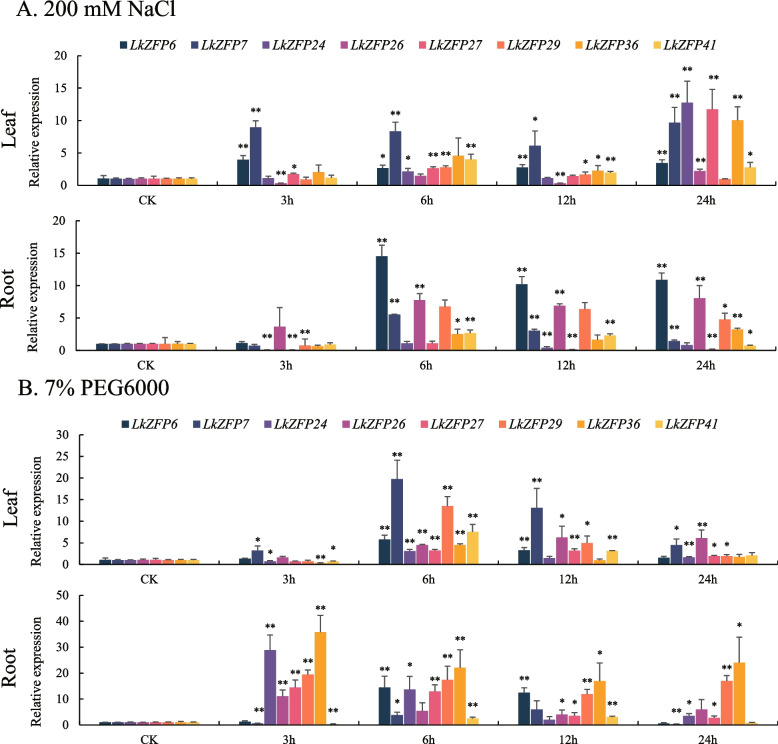
The relative expression level of eight *LkZFP* genes under salt and drought treatment by RT-qPCR. in leaves and rootsError bars represent the deviations from three biological replicates. The standard deviation was shown at the top of the bar chart, and the asterisk indicated significant differences at *P* < 0.05 (*), *P* < 0.01 (**)

After treated with 200 mM of NaCl for 24 h, *LkZFP6*, *LkZFP7*, *LkZFP24*, *LkZFP26*, *LkZFP27, LkZFP29*, *LkZFP36* and *LkZFP41* in the leaf and root showed different expression pattens as follows. In the leaf, *LkZFP6*, *LkZFP7*, *LkZFP24*, *LkZFP27, LkZFP29*, *LkZFP36* and *LkZFP41* were induced by NaCl treatment. *LkZFP6* and *LkZFP7* was significantly up-regulated at all time points. *LkZFP26* was significantly down-regulated at 3 h and 12 h. *LkZFP7*, *LkZFP24*, *LkZFP26*, *LkZFP27* and *LkZFP36* reached their highest level after 24 h of treatment. *LkZFP29* and *LkZFP41* reached their highest level after 6 h, and *LkZFP29* was comparable to untreated control at 24 h (Fig. 7A). In the root, *LkZFP6*, *LkZFP7*, *LkZFP24*, *LkZFP27*, *LkZFP29*, *LkZFP36* and *LkZFP41* were inhibited after 3 h of treatment, reached the highest level after 6 h and then gradually decreased at the following time points. However, *LkZFP6* and *LkZFP26* were up-regulated at all time points (Fig. 7A).

After treated with 7% PEG6000 in the leaf, *LkZFP24*, *LkZFP36* and *LkZFP41* were significantly down-regulated at 3 h. *LkZFP6*, *LkZFP7*, *LkZFP24*, *LkZFP27*, *LkZFP29*, *LkZFP36* and *LkZFP41* were up-regulated to the maximum at 6 h, and then gradually decreased. *LkZFP26* was up-regulated at all time points (Fig. 7B). In the root, *LkZFP24*, *LkZFP26*, *LkZFP27*, *LkZFP29* and *LkZFP36* were induced by drought treatment. *LkZFP36* showed the highest expression among eight Q-type *LkZFP* genes. The expression of *LkZFP7* and *LkZFP41* reached their highest level after 6 h and 12 h treatment, and lower than that of the untreated control group at 3 h and 24 h (Fig. 7B).

**Fig. 8:**
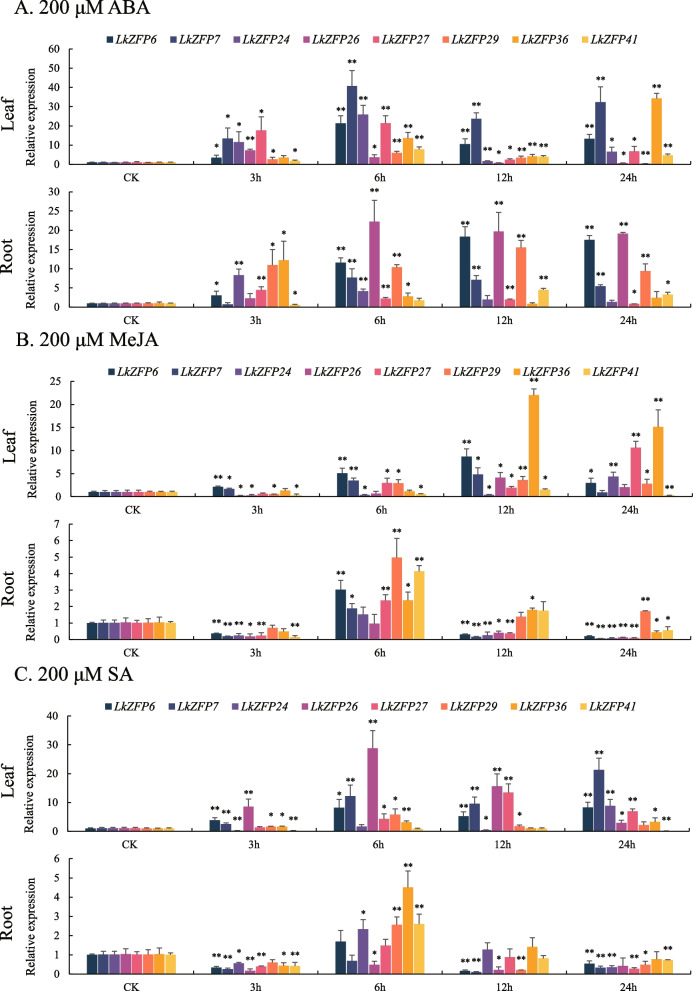
The relative expression level of eight *LkZFP* genes under ABA, MeJA and SA treatment by RT-qPCR. Error bars represent the deviations from three biological replicates. The standard deviation was shown at the top of the bar chart, and the asterisk indicated significant differences at *P* < 0.05 (*), *P* < 0.01 (**)

After treated with 200 μM of ABA in the leaf, *LkZFP36* reached the highest level after 24 h treatment, *LkZFP6*, *LkZFP7, LkZFP24*, *LkZFP27*, *LkZFP29* and *LkZFP41* reached the highest level at 6 h. *LkZFP29* was significantly down-regulated at 24 h. The expression of *LkZFP26* was significantly up-regulated and the highest at 3 h, gradually decreased after 6 h, and significantly down-regulated at 12 h and 24 h (Fig. 8A). In the root, *LkZFP6*, *LkZFP24*, *LkZFP26* and *LkZFP29*were up-regulated at all time points. At 3 h of treatment, *LkZFP7* and *LkZFP41* was down-regulated. The expression level of *LkZFP24*, *LkZFP27* and *LkZFP36* was up-regulated to the maximum at 3 h, and then gradually decreased (Fig. 8A).

After treated with 200 μM of MeJA in the leaf, *LkZFP6*, *LkZFP7*, *LkZFP26*, *LkZFP29*, *LkZFP36* and *LkZFP41* reached the highest level at 12 h, *LkZFP24* and *LkZFP27* reached the highest level at 24 h. *LkZFP36* showed the highest expression among Q-type *LkZFP* genes. *LkZFP24* and *LkZFP41* were significantly down-regulated after 3 h and 6 h. *LkZFP26* and *LkZFP29* were only significantly down-regulated after 3 h. *LkZFP41* was significantly down-regulated after 3 h, 6 h and 24 h (Fig. 8B). In the root, *LkZFP6*, *LkZFP7*, *LkZFP24*, *LkZFP26*, *LkZFP27*, *LkZFP29*, *LkZFP36* and *LkZFP41* reached the highest level at 6 h and were inhibited at most of the time. And *LkZFP6*, *LkZFP7*, *LkZFP24*, *LkZFP26*, *LkZFP27*, *LkZFP36* and *LkZFP41* were significantly down-regulated at 24 h (Fig. 8B).

After treated with 200 μM of SA in the leaf, *LkZFP6*, *LkZFP7*, *LkZFP26*, *LkZFP27*, *LkZFP29* and *LkZFP36* were up-regulated at all time points, *LkZFP41* were down-regulated at all time points. *LkZFP24* was significantly down-regulated after 3 h and 12 h of treatment (Fig. 8C). In the root, *LkZFP6*, *LkZFP24*, *LkZFP27*, *LkZFP29*, *LkZFP36* and *LkZFP41* were up-regulated at 6 h. *LkZFP7* and *LkZFP26* were down-regulated at all time points. *LkZFP6*, *LkZFP7*, *LkZFP24*, *LkZFP27* and *LkZFP41* were significantly down-regulated after 3 h and 24 h (Fig. 8C).

### Subcellular localization

**Fig. 9:**
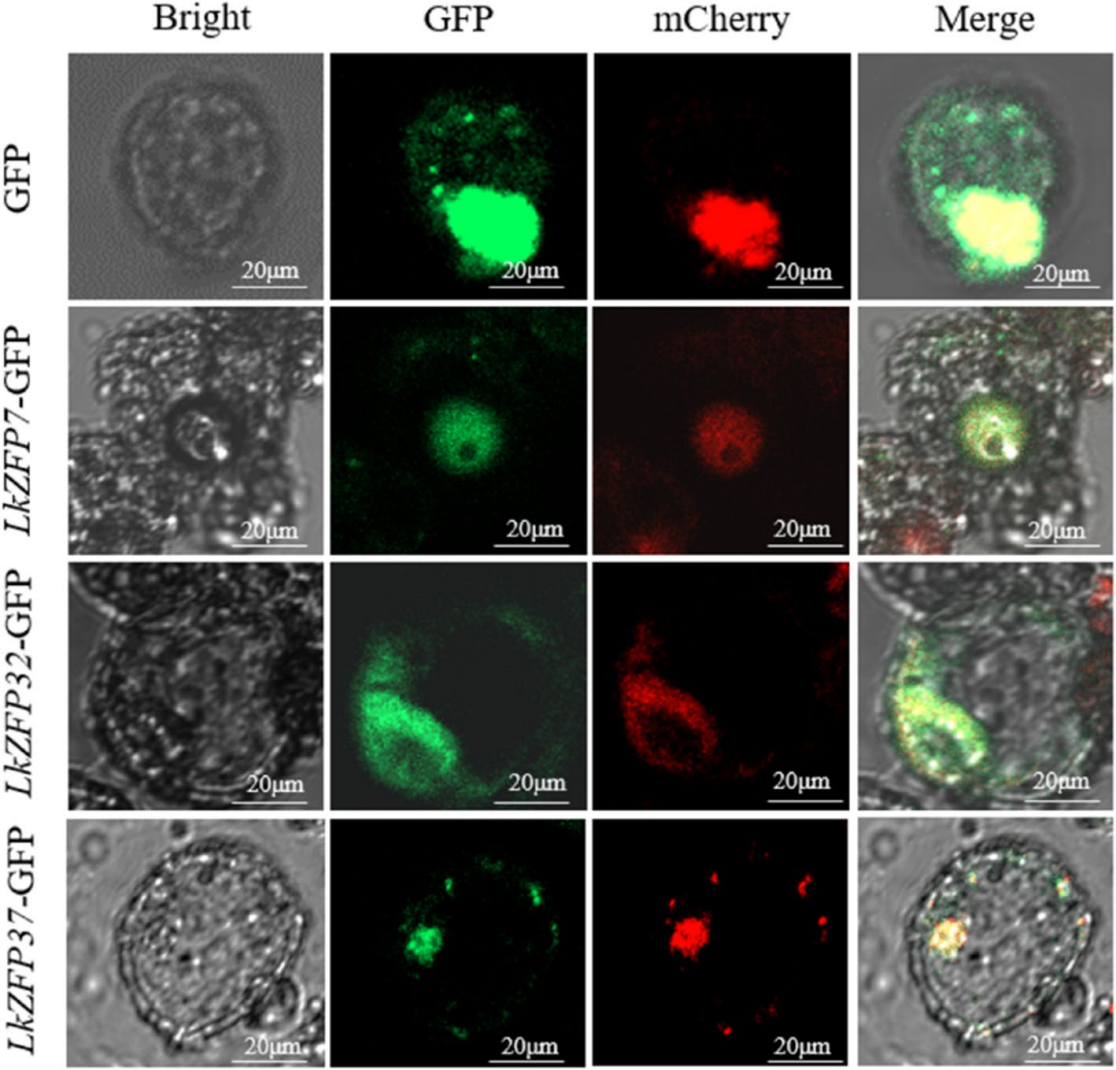
Subcellular localization of *LkZFP7*, *LkZFP32* and *LkZFP37* in “Yinzhong” Qu 2 protoplasts. Bright, green fluorescent protein (GFP), mCherry, and merge are shown. Scale bar = 20 μm. The 35Spro::GFP fusion protein was used as positive control protein

To verify the prediction of subcellular localization using online tool WoLF PSORT, we randomly selected *LkZFP7*, *LkZFP32* and *LkZFP37* and we transformed the GFP fusion vector (35Spro::*LkZFP7*-GFP, 35Spro::*LkZFP32*-GFP and 35Spro::*LkZFP37*-GFP) into “Yinzhong” Qu 2 protoplasts. The results of confocal microscopy revealed that showed that 35Spro::GFP, a positive control, showed a green fluorescence signal in both cytoplasm and nucleus. 35Spro::*LkZFP32*-GFP was located in both cytoplasm and nucleus, 35Spro::*LkZFP7*-GFP and 35Spro::*LkZFP37*-GFP were only located in the nucleus (Fig. 9). The subcellular localization results of *LkZFP7*, *LkZFP32* and *LkZFP37* were consistent with the prediction.

## Discussion

C2H2-ZFPs are widely distributed in plants and play an important role in the regulation of various stages of plant growth and development as well as abiotic stress responses [33–35]. Over the past few decades, this gene family has been extensively studied and proved to have different functions in many plants [36–38], but no comprehensive investigation was reported in *L. kaempferi* which has important economic value. In this study, we identified 47 *LkZFPs* with the conserved domain of “X2-C-X (2–4)-C-X12-H–X (3–5)-H”. The length of these sequences varied significantly from 104 to 896 amino acid residues, indicating a high degree of complexity between *LkZFPs*. The structural diversity may reflect different functions in response to signaling pathways in multiple environments [39, 40].

Accurate phylogenetic trees could help us to understand the evolutionary process of genes, and members of the same group generally have the same ZFP domain number and motifs [41, 42]. By combining the analysis of phylogenetic tree and conserved motifs, we found that the type and arrangement of motifs in the same group were very consistent. It illustrated that *LkZFPs* in the same subgroup may have similar biological functions. Many Q-type C2H2-ZFPs play an important role in different environmental stress responses [43, 44]. Among eight Q-type C2H2-ZFPs identified, we found that six *LkZFPs* contain EAR motif at C-terminus. They may be involved in transcriptional inhibition but require further experimental verification [45, 46]. In addition, since the CDS sequences of the third-generation transcriptions of larch were not available, we could not analyze the exon–intron structure of these genes.

It has been widely reported that *cis*-elements in gene promoters play an important role in transcriptional regulation [47, 48]. Analysis of *cis*-elements is helpful to study expression regulation of transcription factors [49]. The analysis results showed that each member of the *LkZFP* genes contained three or four *cis*-elements associated with hormones or environmental stresses, suggesting that they could regulate reaction. Some *LkZFP* genes have both drought and ABA response *cis*-elements. It may indicate these genes may respond to drought stress through the ABA signaling pathway [50], but specific functions need to be confirmed by further research. Through GO analysis, the *LkZFPs* may be involved in varies biological processes, such as stimulus response and biological regulation.

Previous studies have shown that C2H2-ZFP gene expression is affected by tissue differences and abiotic stresses [51, 52]. Moreover, ABA can accumulate up-regulation during drought and salt treatments and resist osmotic stress by inducing the expression of a range of resistance genes [53, 54]. For example, *StZFP1* in potato and *ZFP179* in rice can be induced by salt stress, drought stress and exogenous ABA [55]. *AtAZF2* may respond to stress through an ABA-dependent pathway [56]. According to the results of RT-qPCR, we considered that *LkZFP6*, *LkZFP7*, *LkZFP29*, *LkZFP36* and *LkZFP41* could be induced by ABA treatment, salt and drought stresses, *LkZFP24*, *LkZFP26*, *LkZFP27* and *LkZFP36* showed similar expression patterns after salt and ABA treatments. In *L. kaempferi*, we found that the transcription levels of many *LkZFP* genes increased under different abiotic stress, but the *LkZFP* genes were more sensitive to salt stress, drought stress, and ABA treatment than MeJA and SA treatments. Interestingly, the relative expression levels of *LkZFP24*, *LkZFP27* and *LkZFP36* in the root were significantly higher than those in the leaves under drought stress. Differences in expression patterns suggest that these genes perform different biochemical functions to adapt to complex challenges. The expression patterns of *LkZFP* genes under different abiotic stress will provide many new insights into the resistance mechanism of *L. kaempferi*. Subcellular localization of three *LkZFP*s (*LkZFP7*, *LkZFP32* and *LkZFP37*) demonstrated the accuracy and reliability of the prediction results.

## Conclusion

In this study, we identified 47 *LkZFP* genes from three generations of larch transcription files and performed a comprehensive bioinformatic analysis. The *LkZFP* genes were divided into 4 subfamilies and 10 subgroups by phylogenetic analysis. By conserved motif analysis, EAR motif, transcriptional inhibition domain, was found in six of the eight Q-type C2H2-ZFPs. GO annotation predicted that *LkZFPs* were involved in a variety of biological processes, such as metabolic processes and biological regulation. Based on promoter cis-element and RT-qPCR analysis, some of *LkZFP* genes respond to salt, drought stress, ABA, MeJA, SA treatment. Subcellular localization results showed that *LkZFP7* and *LkZFP37* were located in the nucleus, *LkZFP32* was located in both cytoplasm and nucleus. The results of this study provide a solid foundation for further functional studies of the *LkZFP* gene family.

## Methods

### Data collection and identification of *LkZFPs*

Due to the large size of larch genome files in NCBI, we turned to three generations of larch transcription files as the base database. All 173 C2H2 zinc finger gene sequences of *Arabidopsis thaliana* were downloaded from the Arabidopsis Information Resource (TAIR) (https://www.arabidopsis.org/), regarded as reference sequences and compared in BioEdit 7.0 [57] to acquire similar genes. The Hidden Markov Model (HMM) of C2H2-ZFPs (Pfam ID: PF00096) downloaded from the Pfam database (http://pfam.xfam.org/) [58], and were used to extract the sequences containing conservative domain by the HMMER 3.3.2 (http://hmmer.org/). Then, we detected their structural domains by Batch SMART program of TBtools 1.09 [59] and deleted redundant sequences. Finally, we summarized and gained the *LkZFPs*. The ProtParam tool of ExPASy (https://web.expasy.org/protparam/) [60] was used to predict the physicochemical properties, such as amino acid (aa) length, molecular weight (MW), theoretical isoelectric point (pI), GRAVY, aliphatic index and instability index. WoLF PSORT (https://wolfpsort.hgc.jp/) [61] was used to predict the subcellular localization.

### Phylogenetic analysis

The protein sequences of *L. kaempferi* and *Arabidopsis thaliana* were compared by The ClustalW function of Mega-X 10.0.5 [62]. Then the phylogenetic evolutionary tree was constructed by maximum likelihood estimation (MLE) with 1000 times bootstraps [63]. Furthermore, we used Evolview (https://www.evolgenius.info/evolview-v2) [64] to beautify the phylogenetic tree. The Q-type zinc finger proteins in *L. kaempferi* were aligned by ClustalX 2.0 [65]. The alignment results were mapped and marked with GeneDoc 2.7 to analyze the homologous parts of C2H2-ZFPs.

### Identification of conserved motifs

In order to further explain the evolutionary relationship between the further, the phylogenetic tree of the *LkZFPs* was drawn separately. The protein conserved motifs were searched by MEME (http://meme-suite.org/tools/meme) [66], and the maximum number was set to 10. Then, the evolution tree and conserved motifs of the *LkZFPs* were visualized using TBtools 1.09.

### Promoter *cis*-element analysis and Gene Ontology Annotation analysis

NCBI BLAST was used to find the 2000 bp promoter sequence of the *LkZFPs*, and it was submitted to PlantCARE (http://bioinformatics.psb.ugent.be/webtools/plantcare/html/) for prediction and analysis of *cis*-elements. The results were then visualized with TBtools software. Sequence alignment was plotted using GeneDoc 2.7. EggNOg-Mapper (http://eggNOG-mapper.embl.de) can associate proteins with GO annotations (parameter default), in the terms of biological process, molecular function and cellular component. Then we used TBtools to collate the data and draw.

### Plant materials and stress treatments

The wild-type *L. kaempferi* was grown in pots containing vermiculite and a soil mix of humus in a ratio of 1:1. The seedlings were grown at 23–25℃ culture room with a 16-h photoperiod. After three months of cultivation, we conducted the following treatments. The seedlings were immersed in 1/2 MS liquid medium containing 200 mM NaCl, 7% polyethylene glycol (PEG) 6000, 200 μM ABA, 200 μM MeJA and 200 μM SA for salinity, drought stress and hormone treatments, respectively. The leaves and roots from different seedlings were collected after 3, 6, 12 and 24 h of treatment respectively and the samples without treatment (0 h) were used as the control. All samples were frozen in liquid nitrogen after immediately collected, and then stored at − 80 °C until analysis.

### RNA isolation and RT-qPCR

Total RNA was extracted from *L. kaempferi* leaves and roots using the Plant RNA Reagent Kit (Bioteke, Wuxi, China). Total 1 μg of total RNA was used for the cDNA synthesis by using the MonScript™ RTIII All-in-One Mix with the dsDNase Kit (Monad, Wuhan, China). The synthesized cDNA was diluted ten times for RT-qPCR template and three replicate PCR amplifications were performed for each sample. The α-tubulin gene and actin gene were selected as internal references and Primer Premier 5 was used to design primers with amplicon lengths of 175–221 bp. The primer sequences of *LkZFP* genes are listed in the supplementary material (Table S1). The RT-qPCR used ChamQ Universal SYBR qPCR Master Mix (Vazyme, Nanjing, China). The reaction system consisted of 10µL of 2 × ChamQ Universal SYBR qPCR Master Mix, 0.4µL (10 µM) of forward primer, 0.4µL (10 µM) of reverse primer, 1µL (100 ng) of cDNA, and 8.2µL of ddH_2_O. The reaction process was performed with the following steps: 95℃ for 30 s; forty cycles were performed with 95℃ for 10 s and 60℃ for 30 s. Relative expression levels of *LkZFP*s were determined using the 2^−ΔΔCt^ method [67].

### Subcellular localization analysis

The full-length CDS of randomly selected three genes *LkZFP7*, *LkZFP32* and *LkZFP37* were amplified using specific primers (Table S2) and KOD FX DNA Polymerase (TOYOBO, Osaka, Japan), respectively, and then cloned into plasmids 35Spro::GFP. Protoplasts were extracted following the procedure described previously [68]. The constructed GFP fusion vector (35Spro::*LkZFP7*-GFP, 35Spro::*LkZFP32*-GFP and 35Spro::*LkZFP37*-GFP) were transfected into “Yinzhong” Qu 2 protoplasts [68] and cultured in dark at 25℃ for 16 h. The 35Spro::GFP transfected into the protoplasts as control. The fluorescence signals were observed and collected by a laser scanning confocal microscopy (LSM880, ZEISS, Jena, Germany) [69, 70].

### Data analysis

Statistical testing was performed with IBM SPSS statistical software (version 23). Three biological replicates were set for each sample of experiments. The data were tested by Student’s *t*-test (*P < 0.05 or **P < 0.01).

## Supplementary Information

**Additional file 1:** **Table S1.** Primers used for RT-qPCR analysis. **Table S2.** Primers used for subcellular localization.

## Data Availability

The sequences of *LkZFP7* (OQ630901), *LkZFP32* (OQ630902) and *LkZFP37* (OQ630903) are available in NCBI (https://submit.ncbi.nlm.nih.gov/).

## References

[1] Laity JH, Lee BM, Wright PE (2001). Zinc finger proteins: new insights into structural and functional diversity. Curr Opin Struct Biol.

[2] Franco-Zorrilla JM, López-Vidriero I, Carrasco JL, Godoy M, Vera P, Solano R (2014). DNA-binding specificities of plant transcription factors and their potential to define target genes. Proc Natl Acad Sci U S A.

[3] Miller J, McLachlan AD, Klug A (1985). Repetitive zinc-binding domains in the protein transcription factor IIIA from Xenopus oocytes. EMBO J.

[4] Berg JM, Shi Y (1996). The galvanization of biology: a growing appreciation for the roles of zinc. Science.

[5] Liu Y, Khan AR, Gan Y. C2H2 Zinc Finger Proteins Response to Abiotic Stress in Plants. Int J Mol Sci. 2022;23(5):2730. 10.3390/ijms23052730. Published 2022 Mar 1. 10.3390/ijms23052730 PMC8911255 35269875

[6] Chen Y, Wang G, Pan J, et al. Comprehensive Genomic Analysis and Expression Profiling of the C2H2 Zinc Finger Protein Family Under Abiotic Stresses in Cucumber (Cucumis sativus L.). Genes (Basel). 2020;11(2):171. 10.3390/genes11020171. Published 2020 Feb 6. 10.3390/genes11020171 PMC7074296 32041281

[7] Ciftci-Yilmaz S, Mittler R (2008). The zinc finger network of plants. Cell Mol Life Sci.

[8] Wolfe SA, Nekludova L, Pabo CO (2000). DNA recognition by Cys2His2 zinc finger proteins. Annu Rev Biophys Biomol Struct.

[9] Alam I, Batool K, Cui DL, Yang YQ, Lu YH. Comprehensive genomic survey, structural classification and expression analysis of C2H2 zinc finger protein gene family in Brassica rapa L. PLoS One. 2019;14(5):e0216071. 10.1371/journal.pone.0216071. Published 2019 May 6. 10.1371/journal.pone.0216071 PMC6502316 31059545

[10] Takatsuji H (1999). Zinc-finger proteins: the classical zinc finger emerges in contemporary plant science. Plant Mol Biol.

[11] Faraji S, Rasouli SH, Kazemitabar SK. Genome-wide exploration of C2H2 zinc finger family in durum wheat (Triticum turgidum ssp. Durum): insights into the roles in biological processes especially stress response. Biometals. 2018;31(6):1019–1042. 10.1007/s10534-018-0146-y. 10.1007/s10534-018-0146-y 30288657

[12] Gourcilleau D, Lenne C, Armenise C (2011). Phylogenetic study of plant Q-type C2H2 zinc finger proteins and expression analysis of poplar genes in response to osmotic, cold and mechanical stresses. DNA Res.

[13] Han G, Lu C, Guo J, et al. C2H2 Zinc Finger Proteins: Master Regulators of Abiotic Stress Responses in Plants. Front Plant Sci. 2020;11:115. 10.3389/fpls.2020.00115. Published 2020 Feb 20. 10.3389/fpls.2020.00115 PMC7044346 32153617

[14] Wang K, Ding Y, Cai C, Chen Z, Zhu C (2019). The role of C2H2 zinc finger proteins in plant responses to abiotic stresses. Physiol Plant.

[15] Ciftci-Yilmaz S, Morsy MR, Song L (2007). The EAR-motif of the Cys2/His2-type zinc finger protein Zat7 plays a key role in the defense response of Arabidopsis to salinity stress. J Biol Chem.

[16] Lawrence SD, Novak NG. The remarkable plethora of infestation-responsive Q-type C2H2 transcription factors in potato. BMC Res Notes. 2018;11(1):398. 10.1186/s13104-018-3503-6. Published 2018 Jun 19. 10.1186/s13104-018-3503-6 PMC6011193 29921330

[17] Li Y, Sun A, Wu Q, et al. Comprehensive genomic survey, structural classification and expression analysis of C2H2-type zinc finger factor in wheat (Triticum aestivum L.). BMC Plant Biol. 2021;21(1):380. 10.1186/s12870-021-03016-3. Published 2021 Aug 18. 10.1186/s12870-021-03016-3 PMC8375173 34407757

[18] Takatsuji H, Mori M, Benfey PN, Ren L, Chua NH (1992). Characterization of a zinc finger DNA-binding protein expressed specifically in Petunia petals and seedlings. EMBO J.

[19] Mahapatra M, Mahanty B, Joshi RK (2019). Genome wide identification and functional assignments of C2H2 Zinc-finger family transcription factors in Dichanthelium oligosanthes. Bioinformation.

[20] Han G, Yuan F, Guo J, Zhang Y, Sui N, Wang B (2019). AtSIZ1 improves salt tolerance by maintaining ionic homeostasis and osmotic balance in Arabidopsis. Plant Sci.

[21] Sun B, Zhao Y, Shi S, Yang M, Xiao K. TaZFP1, a C2H2 type-ZFP gene of T. aestivum, mediates salt stress tolerance of plants by modulating diverse stress-defensive physiological processes. Plant Physiol Biochem. 2019;136:127–142. 10.1016/j.plaphy.2019.01.014. 10.1016/j.plaphy.2019.01.014 30665058

[22] Yu Z, Yan H, Liang L, et al. A C2H2-Type Zinc-Finger Protein from Millettia pinnata, MpZFP1, Enhances Salt Tolerance in Transgenic Arabidopsis. Int J Mol Sci. 2021;22(19):10832. 10.3390/ijms221910832. Published 2021 Oct 7. 10.3390/ijms221910832 PMC8509772 34639173

[23] Han YC, Fu CC (2019). Cold-inducible MaC2H2s are associated with cold stress response of banana fruit via regulating MaICE1. Plant Cell Rep.

[24] Yin J, Wang L, Zhao J (2020). Genome-wide characterization of the C2H2 zinc-finger genes in Cucumis sativus and functional analyses of four CsZFPs in response to stresses. BMC Plant Biol..

[25] Englbrecht CC, Schoof H, Böhm S. Conservation, diversification and expansion of C2H2 zinc finger proteins in the Arabidopsis thaliana genome. BMC Genomics. 2004;5(1):39. 10.1186/1471-2164-5-39. Published 2004 Jul 5. 10.1186/1471-2164-5-39 PMC481060 15236668

[26] Liu Q, Wang Z, Xu X, Zhang H, Li C. Genome-Wide Analysis of C2H2 Zinc-Finger Family Transcription Factors and Their Responses to Abiotic Stresses in Poplar (Populus trichocarpa). PLoS One. 2015;10(8):e0134753. 10.1371/journal.pone.0134753. Published 2015 Aug 3. 10.1371/journal.pone.0134753 PMC4523194 26237514

[27] Liu Z, Coulter JA, Li Y, et al. Genome-wide identification and analysis of the Q-type C2H2 gene family in potato (Solanum tuberosum L.). Int J Biol Macromol. 2020;153:327–340. 10.1016/j.ijbiomac.2020.03.022. 10.1016/j.ijbiomac.2020.03.022 32145229

[28] Arrey-Salas O, Caris-Maldonado JC, Hernández-Rojas B, Gonzalez E. Comprehensive Genome-Wide Exploration of C2H2 Zinc Finger Family in Grapevine (Vitis vinifera L.): Insights into the Roles in the Pollen Development Regulation. Genes (Basel). 2021;12(2):302. 10.3390/genes12020302. Published 2021 Feb 20. 10.3390/genes12020302 PMC7924211 33672655

[29] Han XM, Chen QX, Yang Q, Zeng QY, Lan T, Liu YJ (2019). Genome-wide analysis of superoxide dismutase genes in Larix kaempferi. Gene.

[30] Sun C, Xie YH, Li Z (2022). The Larix kaempferi genome reveals new insights into wood properties. J Integr Plant Biol.

[31] Kazan K (2006). Negative regulation of defence and stress genes by EAR-motif-containing repressors. Trends Plant Sci.

[32] Kagale S, Rozwadowski K (2011). EAR motif-mediated transcriptional repression in plants: an underlying mechanism for epigenetic regulation of gene expression. Epigenetics.

[33] Kiełbowicz-Matuk A (2012). Involvement of plant C(2)H(2)-type zinc finger transcription factors in stress responses. Plant Sci.

[34] Muthamilarasan M, Bonthala VS, Mishra AK (2014). C2H2 type of zinc finger transcription factors in foxtail millet define response to abiotic stresses. Funct Integr Genomics.

[35] Lyu T, Liu W, Hu Z, et al. Molecular characterization and expression analysis reveal the roles of Cys2/His2 zinc-finger transcription factors during flower development of Brassica rapa subsp. chinensis. Plant Mol Biol. 2020;102(1–2):123–141. 10.1007/s11103-019-00935-6. 10.1007/s11103-019-00935-6 31776846

[36] Liao X, Wang L, Zhu S, Zheng F, Yang C (2021). Identification, genomic organization, and expression profiles of single C2H2 zinc finger transcription factors in tomato (Solanum lycopersicum). J Appl Genet.

[37] Yang S, Wang Y, Zhu H (2022). A novel HD-Zip I/C2H2-ZFP/WD-repeat complex regulates the size of spine base in cucumber. New Phytol.

[38] Li H, Yue M, Jiang L, et al. Genome-Wide Identification of Strawberry C2H2-ZFP C1–2i Subclass and the Potential Function of FaZAT10 in Abiotic Stress. Int J Mol Sci. 2022;23(21):13079. 10.3390/ijms232113079. Published 2022 Oct 28. 10.3390/ijms232113079 PMC9654774 36361867

[39] Razin SV, Borunova VV, Maksimenko OG, Kantidze OL (2012). Cys2His2 zinc finger protein family: classification, functions, and major members. Biochemistry (Mosc).

[40] Vatansever R, Filiz E, Eroglu S (2017). Genome-wide exploration of metal tolerance protein (MTP) genes in common wheat (Triticum aestivum): insights into metal homeostasis and biofortification. Biometals.

[41] Takahashi H, Buchner P, Yoshimoto N, Hawkesford MJ, Shiu SH (2012). Evolutionary relationships and functional diversity of plant sulfate transporters. Front Plant Sci..

[42] Kapli P, Yang Z, Telford MJ (2020). Phylogenetic tree building in the genomic age. Nat Rev Genet.

[43] Agarwal P, Arora R, Ray S (2007). Genome-wide identification of C2H2 zinc-finger gene family in rice and their phylogeny and expression analysis. Plant Mol Biol.

[44] Wang F, Tong W, Zhu H (2016). A novel Cys2/His2 zinc finger protein gene from sweetpotato, IbZFP1, is involved in salt and drought tolerance in transgenic Arabidopsis. Planta.

[45] Kam J, Gresshoff PM, Shorter R, Xue GP (2008). The Q-type C2H2 zinc finger subfamily of transcription factors in Triticum aestivum is predominantly expressed in roots and enriched with members containing an EAR repressor motif and responsive to drought stress. Plant Mol Biol.

[46] Kagale S, Links MG, Rozwadowski K (2010). Genome-wide analysis of ethylene-responsive element binding factor-associated amphiphilic repression motif-containing transcriptional regulators in Arabidopsis. Plant Physiol.

[47] Bernard V, Brunaud V, Lecharny A. TC-motifs at the TATA-box expected position in plant genes: a novel class of motifs involved in the transcription regulation. BMC Genomics. 2010;11:166. 10.1186/1471-2164-11-166. Published 2010 Mar 12. 10.1186/1471-2164-11-166 PMC2842252 20222994

[48] Nakashima K, Yamaguchi-Shinozaki K, Shinozaki K. The transcriptional regulatory network in the drought response and its crosstalk in abiotic stress responses including drought, cold, and heat. Front Plant Sci. 2014;5:170. 10.3389/fpls.2014.00170. Published 2014 May 16. 10.3389/fpls.2014.00170 PMC4032904 24904597

[49] Sharma N, Russell SD, Bhalla PL, Singh MB (2011). Putative cis-regulatory elements in genes highly expressed in rice sperm cells. BMC Res Notes.

[50] Lim CW, Baek W, Jung J, Kim JH, Lee SC. Function of ABA in Stomatal Defense against Biotic and Drought Stresses. Int J Mol Sci. 2015;16(7):15251–15270. 10.3390/ijms160715251. Published 2015 Jul 6. 10.3390/ijms160715251 PMC4519898 26154766

[51] Xiao J, Hu R, Gu T (2019). Genome-wide identification and expression profiling of trihelix gene family under abiotic stresses in wheat. BMC Genomics.

[52] Ding Q, Zhao H, Zhu P, Jiang X, Nie F, Li G. Genome-wide identification and expression analyses of C2H2 zinc finger transcription factors in Pleurotus ostreatus. PeerJ. 2022;10:e12654. 10.7717/peerj.12654. Published 2022 Jan 5. 10.7717/peerj.12654 PMC8742544 35036086

[53] Dong T, Park Y, Hwang I (2015). Abscisic acid: biosynthesis, inactivation, homoeostasis and signalling. Essays Biochem.

[54] Ullah A, Manghwar H, Shaban M (2018). Phytohormones enhanced drought tolerance in plants: a coping strategy. Environ Sci Pollut Res Int.

[55] Tian ZD, Zhang Y, Liu J, Xie CH (2010). Novel potato C2H2-type zinc finger protein gene, StZFP1, which responds to biotic and abiotic stress, plays a role in salt tolerance. Plant Biol (Stuttg).

[56] Sakamoto H, Maruyama K, Sakuma Y (2004). Arabidopsis Cys2/His2-type zinc-finger proteins function as transcription repressors under drought, cold, and high-salinity stress conditions. Plant Physiol.

[57] Hall TA (1999). BioEdit: a user-friendly biological sequence alignment editor and analysis program for Windows 95/98/NT. In Nucleic Acids Symp Ser.

[58] El-Gebali S, Mistry J, Bateman A (2019). The Pfam protein families database in 2019. Nucleic Acids Res.

[59] Chen C, Chen H, Zhang Y (2020). TBtools: An Integrative Toolkit Developed for Interactive Analyses of Big Biological Data. Mol Plant.

[60] Gasteiger E, Gattiker A, Hoogland C, Ivanyi I, Appel RD, Bairoch A (2003). ExPASy: The proteomics server for in-depth protein knowledge and analysis. Nucleic Acids Res.

[61] Horton P, Park KJ, Obayashi T, et al. WoLF PSORT: protein localization predictor. Nucleic Acids Res. 2007;35(Web Server issue):W585-W587. 10.1093/nar/gkm259. 10.1093/nar/gkm259 PMC1933216 17517783

[62] Kumar S, Stecher G, Li M, Knyaz C, Tamura K (2018). MEGA X: Molecular Evolutionary Genetics Analysis across Computing Platforms. Mol Biol Evol.

[63] Bryant C, Fischer M, Linz S, Semple C (2017). On the quirks of maximum parsimony and likelihood on phylogenetic networks. J Theor Biol.

[64] Subramanian B, Gao S, Lercher MJ, Hu S, Chen WH (2019). Evolview v3: a webserver for visualization, annotation, and management of phylogenetic trees. Nucleic Acids Res.

[65] Larkin MA, Blackshields G, Brown NP, et al. Clustal W and Clustal X version 2.0. Bioinformatics. 2007;23(21):2947–2948. 10.1093/bioinformatics/btm404. 10.1093/bioinformatics/btm404 17846036

[66] Bailey TL, Boden M, Buske FA, et al. MEME SUITE: tools for motif discovery and searching. Nucleic Acids Res. 2009;37(Web Server issue):W202-W208. 10.1093/nar/gkp335. 10.1093/nar/gkp335 PMC2703892 19458158

[67] Rao X, Huang X, Zhou Z, Lin X (2013). An improvement of the 2-ΔΔCT method for quantitative real-time polymerase chain reaction data analysis. Biostatistics Bioinformatics Biomathematics.

[68] Liu C, Li K, Wang M (2021). Qu-2, a robust poplar suspension cell line for molecular biology. For Res.

[69] Zhao M, Xuan L, Qi H, Shen T, Xu M (2021). Molecular Cloning, Transcriptional Profiling, Subcellular Localization, and miRNA-Binding Site Analysis of Six SCL9 Genes in Poplar. Plants (Basel).

[70] Liu S, Xuan L, Xu LA, Huang M, Xu M. Molecular cloning, expression analysis and subcellular localization of four DELLA genes from hybrid poplar. Springerplus. 2016;5(1):1129. 10.1186/s40064-016-2728-x. Published 2016 Jul 19. 10.1186/s40064-016-2728-x PMC4951394 27478746

