# Supplementary material for: Identification of C2H2 zinc finger genes through genome-wide association study and functional analyses of LkZFPs in response to stresses in Larix kaempferi
Source: BMC Plant Biol. 2023 Jun 2;23:298. doi: 10.1186/s12870-023-04298-5 (PMC10236708; doi:10.1186/s12870-023-04298-5)
Supplement: Supplementary file 1 — Additional file 1: Table S1. Primers used for RT-qPCR analysis. Table S2. Primers used for subcellular localization. [file 12870_2023_4298_MOESM1_ESM.docx]

**Table S1** Primers used for RT-qPCR analysis.

| Gene name | Forward primer (5'-3') | Reverse primer (5'-3') |
| --- | --- | --- |
| *LkZFP6* | GCTGCTTCTGCTTCTGCTTCTG | GCCACCTCCTCTCCCATTGAGG |
| *LkZFP7* | GAGCACAGATTCAGGCGAGCAG | CGCCATGACAGGTGAAGGGAAC |
| *LkZFP24* | TCAGGCGCTGGAGCTGTTCAAG | GCTACCACTTTCACCAGGCTCTAC |
| *LkZFP26* | GGCTAGAAGGGCACAGAGAATG | CCATGAACCTAACCATTGACCATCC |
| *LkZFP27* | CAAACGAGCGCACCTGCTGGC | CGGGATTGATGGACCTGCAAC |
| *LkZFP29* | TGACGATGAAGGAGGAGGTATTGT | AGATTCAGGGTAAGTGGTGGGATG |
| *LkZFP36* | CGCAGTGGCACCTACAACCTTG | GCCCTGATCGGTTCTCGGTTAC |
| *LkZFP41* | ACAAGCGAGAACGCACCTTGGC | GCTCCGCATAAGGTGGCTTGTG |
| α-tubulin | GCCCTCAAATATGCCATGGGC | AATTCAGGGCCGTCTGATGC |
| actin | AGAAATCCAGCCCCTTGTA | CCCCATACCAACCATCACA |

**Table S2** Primers used for subcellular localization.

| Gene name | Forward primer (5'-3') | Reverse primer (5'-3') |
| --- | --- | --- |
| *LkZFP7* | ATGCCGTCCAAGCGACAAAGG | TTAGGATCTTGTAGGACCAAG |
| *LkZFP32* | ATGGATATTATGTTTTCTAAGCC | TTATATTCCACCATGTTTGGCTGC |
| *LkZFP37* | ATGGGAACTCCAGAATTTCCTG | TCAGGGAAGTGAAGTTCCTC |
